# Relationship between Performance on the Mini-Mental State Examination Sub-Items and Activities of Daily Living in Patients with Alzheimer’s Disease

**DOI:** 10.3390/jcm9051537

**Published:** 2020-05-20

**Authors:** Gwanghee Han, Michio Maruta, Yuriko Ikeda, Tomohisa Ishikawa, Hibiki Tanaka, Asuka Koyama, Ryuji Fukuhara, Shuken Boku, Minoru Takebayashi, Takayuki Tabira

**Affiliations:** 1 Doctoral Program of Clinical Neuropsychiatry, Graduate School of Health Sciences, Kagoshima University, Kagoshima 890-8544, Japan; m.maru0111@gmail.com; 2 Department of Neuropsychiatry, Kumamoto University Hospital, Kumamoto 860-8556, Japan; tom.ishikawa.kmm@gmail.com (T.I.); gatapishi0927@gmail.com (H.T.); fryuji@kumamoto-u.ac.jp (R.F.); boku.shuken@kuh.kumamoto-u.ac.jp (S.B.); mtakebayasi@kumamoto-u.ac.jp (M.T.); 3 Department of Rehabilitation, Medical Corporation Sanshukai, Okatsu Hospital, Kagoshima 890-0067, Japan; 4 Department of Clinical Neuropsychiatry, Graduate School of Health Sciences, Kagoshima University, Kagoshima 890-8544, Japan; yuriko@health.nop.kagoshima-u.ac.jp; 5 Department of Neuropsychiatry, Faculty of Life Sciences, Kumamoto University, Kumamoto 860-8556, Japan; asuka@kuh.kumamoto-u.ac.jp; 6 Division of Psychiatry and Neuroscience, Institute for Clinical Research, National Hospital Organization, Kure Medical Center and Chugoku Cancer Center, Hiroshima 737-0023, Japan

**Keywords:** Alzheimer’s disease, mini-mental state examination, activities of daily living

## Abstract

Mini-mental state examination (MMSE) subitems provide useful information about the cognitive status of patients with Alzheimer’s disease (AD). If the relationship between MMSE subitems and activities of daily living (ADL) can be shown, the performance of sub-items can predict ADL status and may provide useful information for early ADL intervention. Therefore, the purpose of this study was to investigate the relationship between MMSE subitem scores and ADL. The study sample consisted of 718 patients with AD. Logistic regression analysis using the Physical Self-maintenance Scale (PSMS) and Lawton’s Instrumental ADL (L-IADL) was performed with each of the subitems as the dependent variables and the MMSE subitem as the independent variable. As a result, the subitems of MMSE, which are strongly related to each item in PSMS differed (e.g., toilet: registration odds ratio 3.00, grooming: naming 3.66). In the case of L-IADL, most items were strongly associated with “writing” (e.g., shopping: odds ratio 4.29, laundry 3.83). In clinical practice, we often focus only on the total MMSE score in patients with AD. However, the relationship between each MMSE subitem and ADL suggested in this study may be useful information that can be linked to ADL care from the performance of the MMSE subitem.

## 1. Introduction

According to a 2015 estimate by the International Alzheimer’s Disease Association, over 9.9 million new cases of dementia are diagnosed globally every year—an average of one new case every 3.2 s. The number of people with dementia worldwide was estimated to be 46.8 million in 2015; it is anticipated to reach 74.7 million by 2030 and 131.5 million by 2050 [1]. Alzheimer’s disease (AD) is the most common cause of dementia, accounting for an estimated 60–80% of all cases [2]. Disabilities caused by AD can be divided into behavioral and psychological disorders, cognitive dysfunctions represented by memory impairments, and functional decline. Among these three related disorders, functional decline is the most basic and persistent among patients with AD.

In describing AD, the Diagnostic and Statistical Manual of Mental Disorders (DSM-V) focuses on functional decline [3], which is mainly assessed by “functional ability” or “activities of daily living (ADL)”. There are two types of ADL—“basic” ADL (BADL) [4], and “instrumental” ADL (IADL) [5]. BADL includes basic daily activities such as feeding, changing clothes, and bathing. The IADL encompasses an individual’s ability to adapt to the social environment and functions of society, such as shopping, use of transport, and financial management. Many previous studies have reported that BADL and IADL are affected by cognitive function [6].

Mini-mental state examination (MMSE) is the world’s most widely used tool for assessing cognitive function in both clinical practice and research settings. The MMSE total score correlates with disease progression [7]. In addition, many studies have reported the relationship between MMSE and BADL and IADL [8]. However, most studies examine the relationship between MMSE total scores and BADL and IADL. The sub-items of the MMSE are used to describe a wide range of cognitive functions, including attention, memory, verbal ability, and visuospatial cognitive function [9]. Although cognitive impairment in AD primarily affects memory in the early stages, other aspects of cognitive function (such as attention, language, visuospatial cognitive function, and executive function) are also affected during disease progression. The various subitems of the MMSE provide useful information about the order in which individual aspects of cognition begin to decline and the progression of AD. Prakoso et al. [10] examined the association between the MMSE subitem and IADL in post-stroke patients and reported that “orientation to time” was associated with “responsibility for own medications” and “ability to handle finances”. In Huisingh et al. [11], the MMSE sub-items were used to investigate the factors that contribute to the risk of driving accidents among the elderly. They dichotomized the MMSE subitem as follows: “orientation to time” (0–3 vs. 4–5 points), “orientation to place” (0–3 vs. 4–5 points), “registration” (0–2 vs. 3 points), “calculation” (0–3 vs. 4–5 points), “recall” (0–2 vs. 3 points), “language” (0–4 vs. 5–8 points), and “copying” (0 vs. 1 point). They reported an association between “orientation to place” and elderly car accidents. From these facts, it is possible to obtain useful information not only from the total points of the MMSE, but also from the subitems of the MMSE. However, in the above prior study, the MMSE “3-step command”, “repeating”, “naming”, “writing”, and “reading and obeying” were collectively investigated as linguistic domains. The “3-step command”, “repeating”, “naming”, “writing”, and “reading and obeying” are different evaluations of cognitive function, especially “writing” because it evaluates multiple cognitive functions [12]. In addition, “3-step command”, “repeating”, “naming”, “writing”, and “reading and obeying” are different in that they decrease with the progress of AD [13]. Therefore, examining the relationship with ADL separately for each MMSE subitem can provide more specific information and may be useful information in actual clinical practice. However, no studies have examined the association of MMSE subitems with BADL and IADL in patients with AD. Hall et al. [14] reported that these cognitive assessments were related to BADL and IADL using Wechsler logic memory, Clock Drawing Test (CDT), and Trail Making Test (TMT) A and B in patients with AD. In their report, Wechsler Logic Memory was associated with “ability to handle finances”, “food preparation”, “shopping”, and “ability to use telephone”. In addition, the CDT reported that it was related to “responsibility for own medications”, “mode of transportation”, “laundry”, “housekeeping”, and “food preparation”. In the case of BADL, CDT was associated with “grooming”, “bathing”, and “feeding”, and TMT-A reported an association with “dressing”. MMSE “copying” is related to TMT-A and B [15] and CDT [16]. In addition, the relationship between temporal orientation and ADL has been reported [17]. From these facts, we can also predict the association between MMSE sub-items (e.g., “orientation to time”, “copying”) and BADL and IADL. However, clinical practice usually tends to focus only on the MMSE total score and cut-off value. MMSE is often used for the initial consultation of patients with AD. Therefore, if the performance of the MMSE sub-item in patients with AD and the relationship with ADL can be known, it is possible that the status of ADL can be predicted from the performance of the MMSE sub-item as soon as possible. In addition, it may provide useful information for designing early interventions to assist patients with AD in becoming independent of ADL. Hence, the purpose of this study was to clarify the association between BADL and IADL with each of the MMSE subitems.

## 2. Materials and Methods

### 2.1. Participants

All procedures followed the clinical study guidelines of the ethics committee of Kumamoto University and were approved by the university’s internal review board. After providing a complete description of all procedures, written informed consent was obtained from all primary caregivers and assent was obtained from all patients. Participants in this study were outpatients with AD and their primary caregivers were recruited from the Dementia-related Disease Medical Center at Kumamoto University Hospital between April 2007 and March 2017. Regarding the ADL status of patients with AD, we collected information by interviewing the primary caregiver, so only patients with AD with reliable information providers were targeted. All patients underwent structural neuroimaging with magnetic resonance imaging or computed tomography of the brain and routine laboratory testing, including tests of B1 and B12 vitamins and thyroid functions. Patients were diagnosed as having AD if they met the National Institute of Neurological and Communicative Disorders and Stroke/AD and Related Disorders Association’s criteria [18]. Clinical diagnoses were made by experienced senior neuropsychiatrists. The following patients were excluded from this study: (1) patients diagnosed with developmental abnormalities, substance abuse disorders, significant neurologic antecedents, serious psychiatric diseases, brain trauma, brain tumors, major neurological precursors, a B1/B12 vitamin deficiency, blindness, parkinsonism, cervical spondylosis, delirium, hypothyroidism epilepsy, or inflammatory diseases (105); (2) patients who do not have reliable information providers, such as those living alone (145); (3) patients lacking data such as MMSE, Physical Self-maintenance Scale (PSMS), or Lawton’s Instrumental ADL (L-IADL) (44); and (4) MMSE total score of 30 points (1). Of the original pool of 1013 patients, 295 were excluded and 718 were included for analysis.

### 2.2. Physical Self-Maintenance Scale (PSMS)

The PSMS, developed by Lawton et al., assesses patients’ functional ability across six categories of BADL: “toileting”, “feeding”, “dressing”, “grooming”, “physical ambulation”, and “bathing”. Complete independence in these tasks is scored as 1 point, and tasks requiring assistance are scored as 0 points. Higher scores reflect higher ADL functionality. Overall scores range from 0 (i.e., low-functioning, dependent) to 6 (i.e., high-functioning, independent) [5]. The PSMS was conducted by acquiring information about patients from their primary caregiver.

### 2.3. Lawton’s IADL (L-IADL)

The L-IADL, devised by Lawton and colleagues, includes the following items: “ability to use telephone”, “shopping”, “food preparation”, “housekeeping”, “laundry”, “mode of transportation”, “responsibility for own medications”, and “ability to handle finances”. For L-IADL, females were scored on all eight items; however, males were not scored on “food preparation”, “housekeeping”, and “laundry”. As above, complete independence is scored as 1 point, and tasks requiring assistance are scored as 0 points. Higher scores reflect higher IADL functioning. Overall scores range from 0 (i.e., low-functioning, dependent) to 8 (i.e., high-functioning, independent) for females and 0–5 for males [5]. The L-IADL was conducted by acquiring information about patients from their primary caregiver.

### 2.4. Mini-Mental State Examination (MMSE)

The MMSE is a simple cognitive function test, developed by Folstein et al. [19]. MMSE scores decrease with age, and patients with less education tend to have lower MMSE scores [20]. The MMSE comprises 11 sub-items that measure varied brain functions. Each item is scored according to the number of subordinate examination items as follows: “orientation to time” (5 points), “orientation to place” (5 points), “registration” (3 points), “calculation” (5 points), “repetition” (1 point), “3-step command” (3 points), “reading and obeying” (1 point), “recall” (3 points), “naming” (2 points), “writing” (1 point), and “copying” (1 point). “Orientation to time”, “orientation to place”, “calculation”, “recall”, “copying”, and “3-step command” are related to attention and working memory [21]. In addition, “copying” requires complex cognitive functions such as visuospatial cognition and praxis functions [16]. “Writing” involves writing sentences and thinking voluntarily; therefore, it requires complex cognitive functions such as language-specific processes, executive functions [12], and visuospatial cognition [22]. “Registration” and “repetition” evaluate verbal abilities and immediate memory [23].

### 2.5. Statistical Analyses

(1) We confirmed the relationship between the total score of PSMS and L-IADL and the total score of MMSE using Spearman’s rank correlation coefficient. (2) We performed univariate analysis (Spearman’s rank correlation coefficient, χ^2^ test) between MMSE subitems and PSMS and L-IADL subitems. In order to standardize the odds ratio in logistic regression analysis, the scores of each sub-item of MMSE were divided into a perfect score group and a non-perfect score group as follows. “Orientation to time” (0–4 vs. 5 points), “orientation to place” (0–4 vs. 5 points), “registration” (0–2 vs. 3 points), “calculation” (0–4 vs. 5 points), “recall” (0–2 vs. 3 points), “3-step command” (0–2 vs. 3 points), “repeating” (0 vs. 1 point), “naming” (0–1 vs. 2 points), “writing” (0 vs. 1 point), “reading and obeying” (0 vs. 1 point), and “copying” (0 vs. 1 point). The perfect score group received 1 point, and the non-perfect score group received 0 points. (3) In logistic regression analysis, each subitem of PSMS (independence: 1 point, requiring assistance: 0 points) was used as each dependent variable. Then, logistic regression analysis was performed using the MMSE subitems significantly related to each subitem of PSMS in the univariate analysis in (2) above as independent variables (excluding correlation coefficients of 0.20 or less). (4) Each subitem of L-IADL (independence: 1 point, requiring assistance: 0 points) was set as each dependent variable. Then, logistic regression analysis was performed using the MMSE subitems significantly related to each L-IADL subitem in the univariate analysis in (2) above as independent variables (excluding correlation coefficients of 0.2 or less). Prior to logistics regression analysis, Spearman’s rank correlation coefficient between MMSE sub-items was performed to check for multicollinearity between MMSE subitems. Then, it was confirmed that the correlation coefficient between MMSE subitems was less than 0.9 (absolute value). All models were adjusted for age, gender, and education. For all analyses, a probability value of *p* < 0.05 was considered significant. Statistical calculations were performed using IBM SPSS Statistics 23.0 (IBM Corp., Armonk, NY, USA).

## 3. Results

### 3.1. Basic Descriptive Statistics and Mean MMSE Scores

Table 1 shows the average clinical scores, demographics, and MMSE scores of participants. The average MMSE score was 19.52. The average score for “reading and obeying” and “naming” for MMSE sub-items was relatively high, but the average score for “recall” was relatively low. Although the average PSMS score was high, the average L-IADL score was observed to decrease in both females and males.

### 3.2. Correlation between Total Score of PSMS and L-IADL and Total Score of MMSE

The total score of PSMS and the total score of MMSE showed a significant correlation (r = 0.370, *p* = 0.000). The total L-IADL score for males and the total MMSE score showed a significant correlation (r = 0.332, *p* = 0.000), and the total L-IADL score for females also showed a significant correlation with the total MMSE score (r = 0.439, *p* = 0.000).

### 3.3. Univariate Analysis of MMSE Sub-Items and PSMS and L-IADL Sub-Items

Table 2 shows the results of the univariate analysis between the subitems of the MMSE and the sub-items of the PSMS. Table 3 shows the results of the univariate analysis between the subitems of the MMSE and the subitems of the L-IADL. The PSMS and L-IADL each subitem differed in the MMSE subitems showing significant association (correlation coefficient greater than 0.20). However, “orientation to time” and “orientation to place” showed significant association with most sub-items of PSMS and L-IADL. In addition, “writing”, “copying”, and “reading and obeying” showed significant association with all items of PSMS and L-IADL. On the other hand, “recall” did not show a significant association for all items.

### 3.4. Relationship between PSMS Score by Logistic Regression Analysis and MMSE Sub-Item

Before performing multivariate analysis, we performed Spearman’s rank correlation coefficient between MMSE subitems to check the effect of multicollinearity. As a result, there was no item showing a high correlation with a correlation coefficient of 0.9 (absolute value) or more between subitems of each MMSE. The omnibus test for each model coefficient was significant at the 0.1% level, and the results that guaranteed the significance of the regression equation were obtained. In addition, the Hosmer-Lemeshow test results for each model had a significance probability exceeding 0.05, and the model fit was guaranteed [24].

Regarding the results of PSMS sub-item logistic regression analysis, only significant (*p* < 0.05) odds ratios are shown in Table 4. MMSE sub-items with higher odds ratios differed by PSMS sub-items. In “toileting”, “registration” showed the highest odds ratio. In “feeding”, “reading and obeying” showed the highest odds ratio. In “dressing”, “orientation to time” showed the highest odds ratio, also in “physical ambulation”, “orientation to time” showed the highest odds ratio. In “grooming”, “naming” showed the highest odds ratio, also in “bathing”, “naming” showed the highest odds ratio. “Copying” showed a significant odds ratio for all items except “physical ambulation”.

### 3.5. Relationship between L-IADL Score by Logistic Regression Analysis and MMSE Sub-Item

Only significant (*p* < 0.05) odds ratio results of the logistic regression analysis of L-IADL subitems are shown in Table 5. The omnibus test for each model coefficient was significant at the 0.1% level, and the results that guaranteed the significance of the regression equation were obtained. In addition, the Hosmer-Lemeshow test results for each model had a significance probability exceeding 0.05, and the model fit was guaranteed [24].

For “mode of transportation”, “copying” showed the highest odds ratio. “Responsibility for own medications” had the highest odds ratio with “orientation to time”. “Ability to use telephone”, “shopping”, “food preparation”, “housekeeping”, “laundry” and “ability to handle finances” had the highest odds ratio for “writing”. In particular, “writing” showed a significant odds ratio for all items.

## 4. Discussion

This study investigated the relationship between MMSE sub-items and sub-items of the PSMS and L-IADL. In the case of PSMS, the strongly related MMSE sub-items differed depending on the contents of the PSMS sub-items. The “toileting” of PSMS was strongly associated with “registration”. “Registration” is an evaluation of immediate memory, and the results of this study suggest that “toileting” in patients with AD is strongly associated with immediate memory evaluated by MMSE. “Feeding” was found to have the strongest association with “reading and obeying”. “Reading and obeying” requires the memory to remember the first instruction, the language comprehension ability to read and understand the instruction sentence, and the ability to execute according to the instruction. Therefore, “reading and obeying” is a MMSE subitem that requires complex cognitive functions along with “copying” and “writing” [25], and the results of this study suggest that it is most relevant to “feeding”. “Grooming” and “bathing” were strongly associated with “naming”. “Naming” is an evaluation that requires recall the name of the displayed objects. Among the subitems of PSMS, “grooming” and “bathing” involve using multiple things (e.g., shaving, makeup tools, nail clippers, soaps, shampoos, etc.) compared to other subitems. Therefore, “grooming” and “bathing” may be strongly related to correct memory and perception of objects as assessed by “naming”. “Physical ambulation” was related to “orientation to time”. “Physical ambulation” is walking ability, and many studies have reported that the walking state of patients with AD is related to working memory [26]. In the MMSE, subitems related to the working memory are “orientation to time”, “orientation to place”, “calculation”, “recall”, “copying”, and “3-step command” [21]. The results of this study suggest that “physical ambulation” is particularly related to “orientation to time”, among the subitems of the MMSE. The relationship between “dressing” and “orientation to time” was also shown, suggesting that the temporal orientation was also strongly related to the change of clothes for patients with AD. In addition, “orientation to time” was also associated with “grooming” and “bathing”, and was also suggested to be associated with all L-IADL sub-items except “laundry”. Venable et al. [17] also performed the temporal orientation test, the temporal disorganization scale, and the test of time passage in patients with AD and reported the relationship between temporal orientation and BADL and IADL, supporting the results of this study. Furthermore, the L-IADL’s “responsibility for own medications” was most associated with “orientation to time”. “Responsibility for own medications” is an item related to the management of daily medication. Previous studies have reported that “orientation to time” was associated with “responsibility for own medications” [10]. The results of this study also suggested that “orientation to time” is related to “responsibility for own medications” in patients with AD. “Mode of transportation” was strongly related to “copying”. “Mode of transportation” is an item to ask whether patients with AD can drive a car or use public transportation. Apolinario et al. [27] reported that visuospatial cognition was most strongly associated with driving ability. The results of this study suggest that car driving and public transport use in patients with AD may require visual-spatial cognition. In addition, “copying” was shown to be associated with most subitems of PSMS and L-IADL. “Copying” is mainly evaluation of visual-spatial cognition, and previous studies have reported that BADL and IADL in patients with AD are associated with visual-spatial cognitive function [28], supporting the results of this study. In addition, “copying” is also an item that requires multiple cognitive functions, such as attention and working memory [16,21], so it may have been suggested to be associated with many subitems of PSMS and L-IADL. On the other hand, L-IADL’s “ability to use telephone”, “shopping”, “food preparation”, “housekeeping”, “laundry”, and “ability to handle finances” were most strongly associated with “writing”. Furthermore, all items in the L-IADL were associated with “writing”. No previous studies have examined the relationship between writing sentences and ADL. Since the previous research categorized and examined “writing” as a language domain, it is considered that the effect of “writing” could not be shown. Since “writing” uses complex cognitive functions such as executive functions and visual-spatial cognitive functions [29], previous studies have reported that “writing” is a clinical marker of dementia [30]. Previous studies have also reported that executive functions and visual-spatial cognitive functions are associated with the IADL [31,32]. Furthermore, “writing” requires thinking voluntarily about sentences. Among the subitems of MMSE, only “writing” is an item that requires spontaneous thinking. In a previous study that measured cerebral blood flow response in each subitem of MMSE, “writing” showed the highest activation in the prefrontal cortex [33]. Moreover, it has been reported that spontaneity in patients with AD is associated with the prefrontal cortex [34]. From these facts, “writing” may be associated with spontaneity. In addition, previous studies have also reported that spontaneity is strongly associated with IADL [35,36]. Therefore, since “writing” has a feature that requires spontaneity and multiple cognitive functions such as executive function and visual-spatial cognitive function, it may have suggested a strong association with IADL.

This study has several limitations. First, as a result of this univariate analysis, “recall” did not correlate with the sub-items of the PSMS and IADL. This result does not mean that recent memory is not related to ADL. Many previous studies have reported that recent memory impairment affects ADL. Memory impairment is a typical symptom of AD, and the “recall” score of patients with AD has been shown to be markedly reduced compared to patients without AD [37]. Therefore, the results of this study are based on the floor effect of the MMSE’s short-term memory evaluation, and it is assumed that it is difficult to show an association between the result of “recall” and ADL. Second, typical symptoms of AD include behavioral and psychological disorders. The behavioral and psychological symptoms of dementia (BPSD) also affect ADL decline in patients with AD [38]. However, this study did not consider BPSD in patients with AD. Therefore, future researchers should investigate the relationship between BPSD and ADL in patients with AD. In addition, further research is needed on the relationship between the MMSE sub-items and BPSD (e.g., relationship between “writing” and apathy).

MMSE is a simple screening of cognitive function, and it is impossible for MMSE alone to clearly show the relationship between ADL and neuropsychological performance. However, it has not yet been clearly established which specific cognitive domain is associated with which specific functional domain. MMSE is the world’s most widely used tool for assessing cognitive function. In clinical and research settings, the focus is often on the MMSE total score alone. However, it is difficult for clinicians and patients and caregivers to understand what MMSE total scores mean for ADL and their respective cognitive impairments and for actual management [39]. The various subitems of the MMSE provide useful information about the progression of AD, such as reducing or maintaining aspects of cognitive function in patients with AD. On the other hand, functional decline has the most basic and lasting effect on reducing the quality of life of patients with AD, so early intervention is required [40]. Hence, MMSE is a cognitive assessment often used for the initial consultation of patients with AD; the association between MMSE subitems and ADL obtained in this study will help with the early detection of patients with AD who need ADL support, and it may provide information that may be useful in formulating early interventions to maintain independence.

## Figures and Tables

**Table 1:** Basic descriptive data.

Characteristic	Mean ± SD	Min	Max
*N* (female/male)	718 (492/226)		
Age (years)	76.78 ± 8.7	51	97
Education (years)	10.95 ± 2.5	6	22
MMSE	19.52 ± 5.3	0	29
Orientation to time	3.06 ± 1.6	0	5
Orientation to place	3.65 ± 1.4	0	5
Registration	2.74 ± 0.6	0	3
Calculation	2.03 ± 1.6	0	5
Repetition	0.76 ± 0.4	0	1
3-step command	2.47 ± 0.8	0	3
Reading and obeying	0.92 ± 0.3	0	1
Recall	0.35 ± 0.7	0	3
Naming	1.94 ± 0.3	0	2
Writing	0.84 ± 0.4	0	1
Copying	0.76 ± 0.4	0	1
PSMS	4.97 ± 1.5	0	6
L-IADL (male)	2.36 ± 1.8	0	5
L-IADL (female)	3.56 ± 2.8	0	8

*N*: number of people; SD, standard deviation; MMSE, Mini-Mental State Examination; PSMS, Physical Self-maintenance Scale; L-IADL, Lawton’s Instrumental Activities of Daily Living Scale.

**Table 2:** Univariate analysis of MMSE sub-items and PSMS sub-items.

	Toileting	Feeding	Dressing	Grooming	Physical A.	Bathing
Orientation to time ^a^	0.19 ***	0.16 ***	0.31 ***	0.27 ***	0.29 ***	0.29 ***
Orientation to place ^a^	0.14 ***	0.14 ***	0.22 ***	0.27 ***	0.24 ***	0.31 **
Registration ^a^	0.25 ***	0.19 ***	0.17 ***	0.20 ***	0.13 ***	0.25 ***
Calculation ^a^	0.14 ***	0.15 ***	0.21 ***	0.23 ***	0.20 ***	0.30 **
Repetition ^b^	9.76 **	11.83 **	9.83 **	20.61 ***	16.36 ***	27.20 ***
3-step command ^a^	0.13 ***	0.09 *	0.22 ***	0.20 ***	0.21 **	0.21 **
Reading and obeying ^b^	7.65 **	40.94 ***	22.48 ***	23.31 ***	19.52 ***	31.91 ***
Recall ^a^	0.00	0.02	0.07	0.07	0.13 **	0.07
Naming ^a^	0.14 **	0.19 ***	0.16 ***	0.24 ***	0.18 ***	0.29 ***
Writing ^b^	19.61 ***	22.22 ***	42.59 ***	35.09 ***	26.26 ***	58.84 ***
Copying ^b^	23.84 ***	26.65 ***	33.80 ***	53.77 ***	20.64 ***	65.66 ***

Physical A., physical ambulation; ^a^ Spearman’s rank correlation coefficient; ^b^ χ^2^ value. * *p* < 0.05; ** *p* < 0.01; *** *p* < 0.001.

**Table 3:** Univariate analysis of MMSE sub-items and L-IADL sub-items.

	AT	SH	FP	HK	LD	MT	RM	AF
OT. ^a^	0.30 ***	0.35 ***	0.29 ***	0.31 ***	0.32 ***	0.30 ***	0.30 ***	0.32 ***
OP. ^a^	0.31 ***	0.26 ***	0.22 ***	0.22 ***	0.32 ***	0.27 ***	0.23 ***	0.22 ***
Reg. ^a^	0.20 ***	0.15 ***	0.12 *	0.13 **	0.26 ***	0.11 **	0.11 **	0.13 **
Cal. ^a^	0.17 ***	0.17 ***	0.16 **	0.11 *	0.23 ***	0.18 ***	0.10 *	0.15 ***
Rep. ^b^	15.22 ***	26.39 ***	3.54	4.12 *	25.06 ***	8.09 **	7.50 **	18.05 ***
3C. ^a^	0.22 ***	0.19 ***	0.12 **	0.15 **	0.16 ***	0.20 ***	0.15 ***	0.20 ***
RO. ^b^	21.13 ***	21.51 ***	6.70 *	10.28 **	21.43 ***	13.28 ***	6.99 **	7.56 **
Re. ^a^	0.05	0.10 *	0.00	0.00	0.08	0.00	0.02	0.04
Na. ^a^	0.16 ***	0.12 **	0.15 **	0.11 *	0.21 ***	0.14 ***	0.16 ***	0.12 **
Wr. ^b^	53.73 ***	58.90 ***	30.94 ***	26.59 ***	60.36 ***	30.81 ***	17.54 ***	27.69 ***
Cop. ^b^	39.26 ***	34.92 ***	19.17 ***	19.99 ***	38.50 ***	39.05 ***	15.16 ***	15.42 ***

AT, ability to use telephone; SH, shopping; FP, food preparation; HK, housekeeping; LD, laundry; MT, mode of transportation; RM, responsibility for own medications; AF, ability to handle finances; OT., orientation to time; OP., orientation to place; Reg., registration; Cal., calculation; Rep., repetition; 3C., 3-step command; RO., reading and obeying; Re., recall; Na., naming; Wr., writing; Cop., copying; ^a^ Spearman’s rank correlation coefficient; ^b^ χ^2^ value; * *p* < 0.05; ** *p* < 0.01; *** *p* < 0.001.

**Table 4:** Relationship between PSMS score by logistic regression analysis and MMSE sub-item.

PSMS	MMSE	B	Wald χ^2^	OR	95% CI
Toileting	Registration	1.10	15.41 ***	3.00	1.73–5.20
	Copying	0.86	9.88 **	2.36	1.38–4.02
	Writing	0.65	4.12 *	1.91	1.02–3.57
Feeding	Reading and obeying	1.53	7.82 **	4.60	1.58–13.40
	Copying	1.28	7.65 **	3.60	1.45–8.93
Dressing	Orientation to time	0.99	10.66 **	2.68	1.48–4.84
	Copying	0.80	11.94 **	2.22	1.41–3.48
	Writing	0.78	8.66 **	2.19	1.30–3.69
	3-step command	0.56	7.54 *	1.76	1.18–2.63
Grooming	Naming	1.30	7.21 **	3.66	1.42–9.44
	Copying	1.17	21.26 ***	3.21	1.96–5.27
	Orientation to time	0.70	3.96 *	2.00	1.01–3.97
Physical ambulation	Orientation to time	0.75	9.19 **	2.12	1.30–3.45
	3-step command	0.57	9.22 **	1.77	1.23–2.57
Bathing	Naming	1.37	7.12 **	3.93	1.44–10.72
	Orientation to time	1.16	6.44 *	3.19	1.30–7.83
	Copying	0.99	13.56 ***	2.68	1.59–4.54
	Orientation to place	0.90	6.19 *	2.47	1.21–5.02
	Writing	0.74	5.07 *	2.09	1.10–3.97
	Registration	0.68	5.09 *	1.97	1.09–3.56

MMSE, Mini-Mental State Examination; PSMS, Physical Self-maintenance Scale; B, unstandardized beta coefficient; CI, confidence interval; OR, odds ratio; * *p* < 0.05; ** *p* < 0.01; *** *p* < 0.001.

**Table 5:** Relationship between L-IADL score by logistic regression analysis and MMSE sub-item.

L-IADL	MMSE	B	Wald χ^2^	OR	95% CI
Ability to use telephone	Writing	1.13	16.35 ***	3.09	1.79–5.34
	Copying	0.82	14.83 ***	2.29	1.50–3.49
	Orientation to time	0.53	6.59 *	1.70	1.13–2.54
	3-step command	0.46	6.71 *	1.59	1.12–2.25
Shopping	Writing	1.46	21.61 ***	4.29	2.32–7.93
	Orientation to time	0.77	14.29 ***	2.16	1.45–3.22
	Copying	0.72	10.49 **	2.05	1.33–3.16
	Repeating	0.46	4.44 *	1.59	1.03–2.45
Food preparation	Writing	1.79	15.74 ***	6.01	2.48–14.58
	Copying	0.68	6.98 **	1.98	1.19–3.29
	Orientation to time	0.64	6.84 **	1.90	1.18–3.08
Housekeeping	Writing	1.24	10.75 **	3.45	1.65–7.22
	Copying	0.66	6.83 **	1.93	1.18–3.17
	Orientation to time	0.65	6.89 **	1.92	1.18–3.11
Laundry	Writing	1.34	14.39 ***	3.83	1.91–7.66
	Orientation to place	0.80	7.71 **	2.24	1.27–3.94
	Registration	0.68	5.07 *	1.98	1.09–3.58
	Copying	0.63	5.69 *	1.87	1.12–3.13
Mode of transportation	Copying	1.03	19.36 ***	2.81	1.77–4.45
	Writing	0.96	10.98 **	2.61	1.48–4.61
	Orientation to time	0.50	5.84 *	1.65	1.10–2.47
	Orientation to place	0.37	3.97 *	1.45	1.01–2.09
Responsibility for own medications	Orientation to time	0.66	11.64 **	1.93	1.32–2.81
	Writing	0.63	4.58 *	1.87	1.05–3.32
	Copying	0.51	5.09 *	1.66	1.07–2.58
	Orientation to place	0.42	5.42 *	1.52	1.07–2.17
Ability to handle finances	Writing	1.04	9.36 **	2.83	1.45–5.52
	Orientation to time	0.86	19.22 ***	2.36	1.61–3.45
	Repeating	0.56	5.48 *	1.75	1.10–2.80

L-IADL, Lawton’s Instrumental Activities of Daily Living Scale; B, unstandardized beta coefficient; CI, confidence interval; OR, odds ratio; * *p* < 0.05; ** *p* < 0.01; *** *p* < 0.001.
